# Food insecurity and oral health in older adults

**DOI:** 10.3389/froh.2024.1400591

**Published:** 2024-10-24

**Authors:** Anwar T. Merchant, Afsaneh Fallahi, Arissa Huda, Matthew Lohman

**Affiliations:** ^1^ Department of Epidemiology and Biostatistics, Arnold School of Public Health, University of South Carolina, Columbia, SC, United States; ^2^ University of North Carolina, Chapel Hill, NC, United States

**Keywords:** food insecurity, hunger, oral health, quality of life, missing teeth

## Abstract

**Introduction:**

Household food insecurity, defined as inconsistent access to sufficient food in a household, affects 1 in 15 individuals over the age of 60 years in the US. In these individuals it is associated with numerous chronic conditions, medication underuse leading to poorly controlled conditions such as diabetes and hypertension, and poor oral health. However, the relationship between food insecurity and oral health is understudied. We therefore evaluated the associations between food insecurity and aspects of oral health in older US adults.

**Methods:**

We prospectively evaluated a subset of participants of the Health and Retirement Study (HRS) who responded to questions evaluating food insecurity in 2013 and a supplemental questionnaire regarding dental health and dental health services in 2018 (*N* = 472).

**Results:**

Approximately 1 in 5 people in our study reported being food insecure in the last year. Food insecurity was correlated with poor oral health-related quality of life scores and more tooth loss. Individuals who were food insecure reported worse self-rated oral health (OR = 2.67), greater odds of losing 8 or more teeth (OR = 2.35), and lower odds of receiving oral care (OR = 0.60) compared to their food secure peers.

**Conclusions:**

Though individuals experiencing food insecurity were likely to have more unmet oral health needs than their peers, they were less likely to seek dental care. To improve the oral health status of this group, in addition to making oral health care more accessible, it may also be necessary to address the social and environmental factors preventing these people from seeking oral health care.

## Introduction

Household food insecurity defined as inconsistent access to sufficient food (1), is associated with limited access to health care including dental care (2–4), and increased consumption of sugar and processed foods (5). It is plausible therefore that food insecurity could be adversely associated with an individual's oral health. Food insecurity may be classified as marginal (food is available but there is anxiety about consistent access), low (food is sufficient but lacks diversity), and very low (food is lacking in quantity, diversity, and quality) (1). A household in the last two categories is classified as being food insecure. Approximately 5.2 million older US adults ≥60 years (1 in 15 older adults) experienced food insecurity, with disproportionate representation of ethnic minorities and disadvantaged segments of society (6). This included 1 in 4 living below the poverty line, 1 in 5 African Americans, and 1 in 8 Hispanic persons. State prevalence of food insecurity in this group ranged from 3% to 12.5% (6). Individuals with food insecurity have higher levels of physical and mental disorders such as hypertension, hyperlipidemia, diabetes (7), pre-diabetes (8), depression (9, 10), and atherosclerotic disease (11); they are also more likely to have uncontrolled diabetes and LDL cholesterol (12), and medication underuse duet to cost concerns (13).

Food insecurity is associated with increased dental caries prevalence (14) particularly in children (15), among whom it is partly attributed to increased consumption of sugar sweetened beverages, poor quality diet, and shopping for groceries at convenience stores (5). Adults and children experiencing food insecurity are also less likely to visit a dentist and more likely to have unmet dental needs (2–4). Children and older adults may find it difficult to access dental care because of cost, lack of transportation, and unstable housing situation (16). These factors have the potential to adversely affect the oral health of children and older individuals, but few studies have evaluated the relationship between food insecurity and oral health in older adults (3, 4, 17). We therefore evaluated the connection between food insecurity and self-reported oral health and health care behaviors among older adults.

## Methods

This retrospective study used data from the Health and Retirement Study (HRS), an ongoing cohort survey of adults aged 51 and older in the United States. The HRS is sponsored by the National Institute on Aging (grant number NIA U01AG009740) and is conducted by the University of Michigan. The core HRS survey is conducted biennially and asks participants to report on a variety of demographic characteristics, health-related events, and financial variables. Detailed descriptions of the HRS core survey questions, sampling design, and sample characteristics are available from previous research (18, 19). To evaluate the status of nutrition, food consumption, and access to food among participants of the core study, a subset of 12,418 HRS core study respondents were randomly selected to participate in the Health Care and Nutrition Study (HCNS) in 2013, and were mailed survey with relevant questions. HCNS respondents who provided data on food security (*n* = 8,021) formed the base sample in the present study. In 2018, approximately 10% of HRS respondents were randomly selected to receive a supplemental questionnaire regarding dental health and dental health services (20). There were 472 randomly selected individuals with complete information on nutrition and oral health to assess the objectives of this study. Because food insecurity and oral health may be influenced by individuals’ cognitive health and residential status, we further excluded those who were living in a nursing home at baseline (*n* = 6) or ever reported a diagnosis of dementia (*n* = 14) leaving an analytic sample of 452 respondents.

### Measures

Food security was assessed using a six-item version of the US Household Food Security Scale (21), as this is validated and is the most widely used instrument to measure food insecurity allowing comparisons with other studies in the literature Questions ask whether anyone in a respondent's household ran out of food, could not afford balanced meals, reduced meal size, skipped meals, ate less, or didn't eat despite hunger because there wasn't enough money for food, and the frequency of those occurrences. Affirmative responses were assigned a score of 1 and were summed for a total score ranging from 0 to 6. Individuals with scores of 2 or greater were considered to have experienced food insecurity.

Oral health was measured in several ways, using questions from the HRS supplemental oral health module.

*Oral health related quality of life (OHRQoL)* was measured following an approach used in previous studies of the HRS dental health module (22), a validated scale that has been used in previous studies to evaluate oral health related quality of life among older individuals. Respondents were asked whether and how frequently they (a) avoided eating foods, (b) had difficulty relaxing, (c) avoided going out, (d) felt self-conscious, or (e) felt pain or distress due to their teeth, mouth, or dentures. Frequency/degree was measured on a 5-point ordinal scale ranging from 1—“never”/”none at all”—to 5—“very often”/”a great deal” (self-consciousness was measured on a 3-point ordinal scale with possible values of never, sometimes, always). Item responses were summed to calculate an overall OHRQoL summary score (range: 0–23). Tooth loss, denture use, self-reported oral health, and dental care utilization have been associated with cognition in older adults and are therefore relevant to this study.

*Tooth loss* and *denture use* were measured via self-report. Respondents were asked to report whether they had lost (a) four or more teeth or (b) all teeth, separately, from their upper and lower jaw. An ordinal variable was created to indicate total overall tooth loss by combining reported tooth loss from both jaws ranging from 0—lost fewer than 4 teeth from both jaws—to 4—lost all teeth from both jaws. A dichotomous variable was also created to indicate whether a respondent reported losing 8 or more total teeth. Likewise, denture use was measured using a binary variable (1 = reported using dentures; 0 = no reported denture use).

Self-reported oral health was measured by asking respondents to rate the condition of their mouth and teeth on an ordinal scale—poor, fair, good, very good, or excellent. Self-reported oral health was dichotomized (1 = fair/poor; 0 = good, very good, excellent).

*Dental care utilization* was measured with a binary variable indicating whether respondents reported seeing a dentist for dental care (including for dentures) in the past two years (1 = yes; 0 = no). *Out-of-pocket dental expenses* during the previous two years were self-reported in dollars and transformed to log-dollars for analyses.

Several demographic and health-related factors that may confound the association between food insecurity and oral health were considered in analyses. Demographic characteristics included age, gender, race (categorized as white, black, other), and years of education attained. Income was measured in dollars as total household income in the previous year including salary, pensions, annuities, social security, and other income sources. Health-related behaviors and conditions included alcohol use (yes/no), smoking history (never, former, and current), and a summary score of eight common chronic conditions (hypertension, diabetes, cancer, lung disease, heart disease, stroke, psychiatric disorder, arthritis).

### Statistical analysis

*Descriptive statistics* of demographic and health characteristics were calculated for the overall analytic sample and separately by food insecurity status. Spearman's correlations were calculated for key continuous or ordinal variables.

*Least squares linear regression models* were fit to evaluate the association between two separate continuous outcomes: OHRQoL and log out-of-pocket dental expenses. Model coefficients estimated the difference in mean outcomes comparing those who experienced food insecurity to those who did not. Logistic regression models were used to estimate the association between food insecurity and binary outcomes: self-rated oral health, lost ≥8 teeth, denture use, and dental care utilization. All models were adjusted for age, gender, education, race, smoking history, alcohol use, and number of comorbid conditions.

#### Sensitivity analysis

To evaluate the extent to which attrition of the analytic sample (*n* = 452) between 2013 (baseline) and 2018 (when oral health was assessed) could affect the results we performed a supplemental analysis using inverse probability weighting (IPW), which is a method to address for possible selection bias resulting from attrition. The results from this analysis were compared with the model evaluating food insecurity and oral health, conditioning on sample availability (i.e., complete case analysis). Briefly, we first fit a probit regression model among the baseline sample (*n* = 8,021) estimating the likelihood of remaining in the cohort in 2018, as determined by participant demographic and health characteristics (i.e., covariates used for adjustment in regression models). Individual predicted probabilities of selection into the analytic were derived from the resulting model. Finally, regression analyses were repeated in the analytic sample, with respondent values weighted by the inverse of their probability of remaining in the study. Estimates from IPW analyses are valid assuming that attrition is explained by covariates included in the model (i.e., missing at random).

## Results

Among 452 persons in the sample, 19.2% reported being food insecure in the last year. Compared to individuals who were not food insecure, individuals reporting food insecurity were younger, predominantly female, non-white, smokers, had more chronic conditions and lower household income (Table 1). Food insecurity was correlated with poor oral health-related quality of life scores (Spearman's r = 0.244, *p*-value <0.001) and more tooth loss (Spearman's r = 0.187, *p*-value = 0.01). Poor oral health-related quality of life scores were correlated with more tooth loss (Spearman's r = 0.297, *p*-value <0.001) (Table 2). Food insecurity was associated with a higher OHRQOL score (indicating worse oral health) (*β*-coefficient = 1.647, *p*-value = 0.000) but was not associated with out-of-pocket dental expenses (*β*-coefficient = −0.306, *p*-value = 0.415) (Table 3). Individuals who were food insecure reported worse self-rated oral health (OR = 2.67, 95% CI 1.51, 4.72), greater odds of losing 8 or more teeth (OR = 2.35, 95% CI 1.20, 4.60), and lower odds of receiving oral care (OR = 0.60, 95% CI 0.34, 1.05) compared to their food secure peers (Table 4). Repeating the analyses reported in Tables 3, 4 by inverse probability weighting to account for selection bias did not materially change the results (Supplementary Tables 1, 2).

**Table 1:** Characteristics by food insecurity status of participants of the health and retirement study with information on food insecurity and oral health between 2013 and 2018.

	Total sample		Food insecure		Not food insecure		*p*-value
	*N* (%) or M (SD)		*N* (%) or M (SD)		*N* (%) or M (SD)		
Sociodemographic factors							
Age (mean, sd)	64.24	9.65	60.05	8.52	65.22	9.65	<.001
Female	263	58.19%	58	66.67%	205	56.16%	.074
Race							<.001
White	348	77.33%	47	54.64%	301	82.69%	
Black	62	13.78%	23	26.74%	39	10.71%	
Other	40	8.89%	16	18.60%	24	6.59%	
Years education (mean, sd)	13.13	3.23	11.05	3.71	12.63	2.89	<.001
Health-related factors							
Drinks alcohol	254	57.08%	31	36.90%	223	61.77%	<.001
Ever smoked	239	53.95%	54	64.29%	185	51.53%	.035
Household income, $(mean, sd)	77,471.1	109,569.5	29,280.81	25,383.16	88,684.35	118,280.4	<.001
Chronic conditions, 0–8 (mean, sd)	0.17	0.43	0.202	0.53	0.16	0.40	<.001

**Table 2:** Spearman's correlation between oral health related variables and food insecurity*.

	Food insecurity	OHRQOL score	Number of missing teeth	Out-of-pocket dental care expenses
Food insecurity	1.00			
OHRQOL score	.244 <0.001	1.00		
Number of missing teeth	0.187 <0.001	0.297 <0.001	1.00	
Out-of-pocket dental care expenses	−0.0039 0.534	0.064 0.311	0.051 0.433	1.00

Food insecurity score 0–6.

* *r* and *p*-value.

**Table 3:** Beta coefficients from multivariable least squares linear regression models between oral health related variables and food insecurity^a^.

Outcome	β-coefficient	95% confidence interval	*p*-value
OHRQOL score	1.647	0.913, 2.381	0.000
Out-of-pocket dental care expenses	−0.306	−1.045, 0.433	0.415

^a^ Binary food insecurity variable adjusted for age, gender, education, race, smoking, alcohol use, number of comorbid conditions.

**Table 4:** Associations from logistic regression models between oral health related variables and food insecurity^a^.

Outcome	Odds ratio (95% confidence interval)	*p*-value
Self-rated oral health (reference group: good/very good/excellent)	2.67 (1.51, 4.72)	0.001
≥8 teeth lost (reference group: <8 teeth lost)	2.35 (1.20, 4.60)	0.012
Denture use (reference group: no denture use)^b^	1.59 (0.87, 2.91)	0.131
Dental care utilization (reference group: did not visit dentist in past 2 years)	0.60 (0.34, 1.05)	0.076

^a^ Binary food insecurity variable adjusted for age, gender, education, race, smoking, alcohol use, number of comorbid conditions.

^b^ Among people missing ≥10 teeth.

## Discussion

Older individuals experiencing food insecurity in the past year had worse oral health measured by the OHRQL scale, poorer self-reported oral health, and more missing teeth. Food insecurity was not correlated with out-of-pocket dental care expenditures possibly because seeking professional dental care was weighed against competing needs (23). In a study conducted among older Americans facing financial hardship, men who reported skipping medications had a lower prevalence of poor self-reported oral health (17), suggesting that individuals in this group were choosing between competing needs. The Supplemental Nutrition Assistance Program (SNAP) is a federal program aimed at reducing hunger, however receiving SNAP benefits was not associated with better oral health (24), Improving access to dental care for low-income adults and removing barriers to seeking dental care may be necessary to improve the oral health of this group (16).

Limitations of our study include the small sample size, a lack of information from oral examination by a dental health professional, and the possibility of residual confounding. The small sample size did not allow us to evaluate possible variation in the associations by states. Annual income was used as a surrogate for socioeconomic status in this study because it is well-measured in the HRS data, strongly predicts food insecurity, and is affected by other correlates of socioeconomic status such as education and occupation. Strengths of the study include the prospective design, use of a nationally representative sample of older adults, use of inverse probability weighting to control for possible selection bias resulting from loss to follow-up, and control for a number of measured confounders. Some associations could remain undetected due to type-2 error.

Our findings are consistent with a study conducted among older Americans linking financial hardship with worse self-reported oral health (17). The higher prevalence of poor oral health among older adults experiencing food insecurity could be due to them not seeking dental care when it is needed. Another reason could be the consumption of sugar sweetened beverages and foods that increase the risk of dental caries, which have been associated with food insecurity. People experiencing food insecurity consume poor quality diets (25), and find it difficult to consume an appropriate diet for their chronic conditions (26). Diets lacking in adequate intakes of fiber and fruits and vegetables are associated with periodontal disease and poorly controlled diabetes, which could contribute to worse oral and general health in this group. Food insecurity among older individuals has been associated with medication non-adherence (27), poor control of chronic conditions (12), and depression (28, 29), which could also contribute to poor oral health.

Poor oral health could also contribute to adverse systemic outcomes. For example, tooth loss has been associated with difficulty in chewing resulting in reduced consumption of fruits and vegetables and higher intakes of less healthy processed foods. Tooth loss is also associated with poor cognition and increased risk of cardiovascular disease and mortality. As individuals with food insecurity have more tooth loss compared to their peers, they may be at higher risk of a variety adverse health outcomes. Indeed, individuals experiencing food insecurity have more multiple chronic conditions (30), heart disease (11), and behavioral health diagnoses compared with their peers (31). Moreover these individuals are more likely to be African American or Hispanic and socially marginalized groups.

Even though individuals experiencing food insecurity are likely to have more unmet oral health needs than their peers, they are less likely to seek dental care. In a qualitative study, Cruz and colleagues identified persistent barriers to seeking dental care which included housing and food insecurity, inadequate public transportation, limited Medicaid dental benefits for low-income adults (16). Qualitative and quantitative studies are needed verify our findings and understand the reasons driving the associations. Improving the oral health status of this group is challenging and may require addressing social determinants of health that contribute to health disparities together with increasing access to dental care.

## Data Availability

Publicly available datasets were analyzed in this study. This data can be found here: https://hrs.isr.umich.edu/about.
