# Supplementary material for: Food insecurity and oral health in older adults
Source: Front Oral Health. 2024 Oct 24;5:1400591. doi: 10.3389/froh.2024.1400591 (PMC11540812; doi:10.3389/froh.2024.1400591)
Supplement: Supplementary file 1 [file Table1.docx]

Supplementary Table 1, Beta coefficients from multivariable least squares models between oral health related variables and food insecurity corrected for selection bias by inverse probability weighting^↑^

|  | β-coefficient | 95% confidence interval | p-value |
| --- | --- | --- | --- |
| OHRQOL score | 1.89 | 0.83, 2.95 | 0.00 |
| Out-of-pocket dental care expenses | -0.348 | -1.505, 0.809 | 0.554 |
|  |  |  |  |

^↑^Binary food insecurity variable adjusted for age, gender, education, race, smoking, alcohol use, number of comorbid conditions

Supplementary Table 2, Associations from logistic regression models between oral health related variables and food insecurity corrected for selection bias by inverse probability weighting ^↑^

|  | Odds ratio (95% confidence interval) | p-value |
| --- | --- | --- |
| Self-rate oral health | 2.67 (1.51, 4.72) | 0.001 |
| ≥10 teeth lost | 2.35 (1.20, 4.60) | 0.012 |
| Denture use^‡^ | 0.68 (0.20, 2.37) | 0.546 |
| Dental care utilization | 0.60 (0.34, 1.05) | 0.076 |

^↑^Binary food insecurity variable adjusted for age, gender, education, race, smoking, alcohol use, number of comorbid conditions

^‡^Among people missing ≥10 teeth
